# Association Between Metabolic and Endocrine Parameters With Visceral Adiposity Index–Defined Adipose Tissue Dysfunction in Young Adult Males

**DOI:** 10.1155/jobe/5233463

**Published:** 2026-03-28

**Authors:** Gabriela Yuri, Natalia Santillana, Liliana Soto, Ana Pereira, Camila Corvalán, Mariana Cifuentes

**Affiliations:** ^1^ Institute of Nutrition and Food Technology (INTA), University of Chile, Santiago, Chile, uchile.cl; ^2^ Advanced Center for Chronic Diseases (ACCDiS), University of Chile, Santiago, Chile, uchile.cl

**Keywords:** body composition, metabolic syndrome, visceral adipose tissue

## Abstract

**Background:**

Overweight and obesity are defined by body mass index (BMI); however, this indicator does not address the wide variability that exists in metabolic health for a given BMI. This largely depends on adipose tissue functionality and location, with larger visceral depots elevating inflammation and cardiometabolic risk. The visceral adiposity index (VAI) provides a closer approach to adipose tissue functionality; however, there are limited studies addressing its ability to identify differences in metabolic health among young adults with excess weight. We evaluated whether low versus high VAI (L‐VAI vs. H‐VAI) groups differ in body composition, endocrine and metabolic indicators, and indices of metabolic risk and adipose tissue function.

**Methods:**

Fifty‐two healthy adult males with BMI ≥ 25 kg/m^2^ were evaluated in this cross‐sectional study. Subjects were classified as L‐VAI or H‐VAI according to the VAI cutoff proposed for adipose tissue dysfunction (> 2.52). Weight, height, arterial blood pressure, body composition, and metabolic/endocrine parameters were compared in L‐VAI versus H‐VAI groups.

**Results:**

Subjects in the H‐VAI group (∼40%) showed greater truncal fat mass and lower adiponectin/leptin ratio (Ad/Lep), as well as greater indices of insulin resistance (HOMA‐IR and triglyceride/glucose) and the metabolic syndrome severity score. Significant positive correlations were found between VAI and the cardiovascular risk and metabolic syndrome–related factors, plasminogen activator inhibitor‐1 (PAI‐1), uric acid, and HOMA‐IR and an inverse association with adiponectin and Ad/Lep.

**Conclusions:**

Our observations indicate that VAI is a useful tool to assess cardiometabolic risk and adipose tissue functionality in young adult males with overweight and obesity.

## 1. Introduction

Obesity is a widespread chronic low‐grade inflammatory condition that reduces life quality and expectancy [1, 2]. Under conditions of positive energy balance, various factors determine whether adipose tissue expansion occurs mainly through hyperplasia (increase in adipocyte number) or hypertrophy (increase in size), including genetic, hormonal, and other biological variables [3]. When physiological adipose tissue expansion (mainly subcutaneous) is exceeded, ectopic fat accumulation—including visceral depots—increases, largely interfering with metabolic organ function [4–6]. Visceral fat tends to grow predominantly through hypertrophy, a process associated with insulin resistance, proinflammatory cytokine secretion, hypoxia, and fibrosis [7–9], and males tend to accumulate more visceral fat than females [10, 11]. Research in the recent decade has focused on a subgroup of people with obesity who do not exhibit overt cardiometabolic abnormalities [8, 12, 13]. Interestingly, it has been observed that these subjects tend to be younger and show a predominance of subcutaneous over visceral fat distribution [14]. Lower cumulative exposure to risk factors in younger individuals may translate into greater metabolic resilience [15, 16]; however, early detection of risk factors is still important [13].

Body mass index (BMI, weight (kg)/height (m) squared) is a commonly used tool to define overweight (≥ 25 and < 30 kg/m^2^) and obesity (≥ 30 kg/m^2^) [17]. Although it is a simple and cost‐effective tool for clinical practice and nutritional assessment in large populations, its utility is debated. One main limitation is that BMI does not address body composition (lean versus fat mass), or body fat distribution (visceral versus subcutaneous), both of which are important determinants of the association between excess weight and cardiometabolic disorders [18, 19]. Moreover, nothing can be inferred from BMI regarding adipose tissue (dys) function, a major aspect that determines obesity‐related morbidity [20]. Without considering these variables, individuals with the same BMI can present a wide range of metabolic health status [17, 21], limiting the value of the assessment for adequate therapeutic decisions.

In response to the need to establish an index that, beyond BMI‐defined overweight or obesity, reflects adipose tissue dysfunction related to metabolic risk, Amato et al. [22] developed the visceral adiposity index (VAI), a sex‐specific mathematical model based on waist circumference (WC), BMI, as well as circulating high‐density lipoprotein (HDL) cholesterol (HDL‐C) and triglycerides (TG). Validation studies have shown that VAI is independently associated with cardio‐ and cerebrovascular events and inversely correlated with insulin sensitivity and the components of the metabolic syndrome [22, 23]. VAI cutoff points have been developed to indicate visceral adipose dysfunction in Caucasian subjects [23]; however, there are limited studies validating these values in other populations. This study aimed to evaluate the ability of the proposed VAI‐based cutoff for adipose tissue functionality to reflect differences in body composition and indicators of metabolic risk and adipose‐secreted factors in a sample of young males with overweight and obesity.

## 2. Materials and Methods

### 2.1. Subjects

Fifty‐two healthy adult males aged 18–25 years with BMI ≥ 25 kg/m^2^ participated in this study. Exclusion criteria included recent weight loss and treatments for medical conditions that can affect metabolic or inflammatory status, such as diabetes, cancer, liver or kidney disease, hypo‐ or hyperthyroidism, and rheumatoid arthritis. Participants were evaluated at the Institute of Nutrition and Food Technology (INTA), Universidad de Chile, between June 2022 and September 2023. All procedures were approved by INTA’s Scientific Ethics Committee (Approval letter N15, 2021), and informed written consent was obtained from all participants.

### 2.2. Study Assessments

Participants came to INTA following an 8–12 h overnight fast. Each subject responded to the International Physical Activity Questionnaire (IPAQ) [24] to estimate their weekly physical activity by calculating the total metabolic equivalents of task (MET, minutes/week). Weight, height, and WC (midpoint between the last rib and the right iliac crest in a horizontal line to the left iliac crest) were assessed barefoot and wearing light clothing, using a portable electronic scale for weight (Seca 770 or 803, precision of 0.1 kg), a portable stadiometer for height (Seca 217, to the nearest 0.1 cm), and measuring tape for WC (Lufkin, Model W606 PM, with 200 cm capacity and 0.1 cm precision). BMI (kg/m^2^) and waist (cm)/height (cm) ratio (WhtR) were calculated. Body composition was assessed using dual‐energy X‐ray absorptiometry (DXA, Lunar Prodigy Bone Densitometer—GE Healthcare). Lean mass, total fat mass, and truncal fat mass are expressed raw or relative to total body weight, or as fat‐free mass index, or fat mass index (FFMI, FMI, [kg]/height [m^2^]). Blood pressure was assessed using an automated sphygmomanometer located in the right arm, after a 5 min rest. Venous blood samples were collected to measure fasting glucose, cholesterol (HDL and total) and TG (colorimetric assay, VITROS Chemistry Products, QuidelOrtho Corporation, CA, USA), and insulin (chemiluminescent assay, VITROS Immunodiagnostic Products) using VITROS XT 7600 Integrated Systems. LDL cholesterol was calculated, according to Friedewald et al. [25]. Circulating adipokines and metabolites related to adipose tissue dysfunction and inflammation (high molecular weight adiponectin, leptin, C‐reactive protein [CRP], and plasminogen activator inhibitor‐1 [PAI‐1]) were assessed by the Human Luminex Discovery Assay Kit (R&D Systems, MN, USA). Uric acid was determined using a commercial colorimetric kit (Cayman Chemical, MI, USA).

### 2.3. Calculated Indices

VAI was determined according to the formula defined for males as follows: VAI = (WC (cm)/(39.68 + (1.88 × BMI))) × (TG (mmol/L)/1.03) × (1.31/HDL (mmol/L)). Cutoff values for detecting metabolic syndrome (> 2.52 for subjects under 30 years) [23] were used to define the L‐VAI and H‐VAI study groups.

Insulin sensitivity was assessed by the Homeostatic Model Assessment of Insulin Resistance (HOMA‐IR), an estimative method that assesses insulin sensitivity and β‐cell function derived from fasting plasma glucose and insulin concentrations [26]: (glucose (mmol/L) × fasting insulin (μU/mL)/22.5). Insulin resistance was also assessed by the triglyceride glucose index (TyG), which is the product of fasting TG and glucose: (Ln [fasting triglycerides (mg/dL) × fasting glucose (mg/dL)/2]) [27].

The metabolic syndrome severity score (MSSS) was formulated as a sex‐ and race‐specific equation to overcome the limitations of assessing metabolic syndrome as a dichotomic variable [28]. As a continuous variable, MSSS shows a strong correlation with long‐term risk of cardiovascular disease and type 2 diabetes [29, 30]. The formula for Hispanic males was used as follows: MSSS = −5.5541 + 0.0135 ∗ WC − 0.0278 ∗ HDL + 0.0054 ∗ SBP + 0.8340 ∗ ln (TG) + 0.0105 ∗ fasting glycemia.

### 2.4. Statistical Analysis

Given the relatively small sample size of our study, we performed Shapiro–Wilk tests to determine the distribution of the variables and the appropriate statistical method. Of 34 variables, 24 showed significant deviation from normality (*p* < 0.05); therefore, we used nonparametric statistical tests. Data are presented as medians and interquartile range (p25–p75). L‐VAI and H‐VAI groups were compared using the Wilcoxon rank‐sum (Mann–Whitney) test, and associations between continuous variables were evaluated using Spearman’s correlation coefficients. Partial Spearman’s correlation via residual analysis was performed to account for potential confounders. *p* values < 0.05 were considered significant. Statistical analysis was performed using Stata Version 16.0 (StataCorp LLC, TX, USA).

## 3. Results

A total of 52 participants aged 21.3 ± 2.0 years were included. Thirty‐two volunteers (61.5%) presented VAI values below the age‐specific cutoff established for adipose tissue dysfunction [23]. Regarding the variables that define VAI, the H‐VAI group showed significantly elevated TG, whereas WC and BMI did not differ significantly between groups (Tables 1 and 2). Even though HDL cholesterol levels were below the recommended cutoff in both groups [31], they were significantly lower in H‐VAI subjects (Table 2). Weekly physical activity as estimated by total METs did not differ between groups, with a median (p25–p75) value of 2.452 (1.078–4.662) MET min/week.

**TABLE 1:** Anthropometric and body composition variables of study participants, total sample, and by VAI cutoff^∗^.

	Total (*n* = 52)	Low VAI (*n* = 32)	High VAI (*n* = 20)	*p*‐value^ⴕ^
Weight (kg)	96.1 (91.1–107.1)	95.4 (90.45–104.7)	98.2 (91.4–114.2)	0.288
Height (cm)	173.8 (170.4–178.4)	173.7 (170.5–179.2)	173.8 (169.9–177.3)	0.510
BMI (kg/m^2^)	31.7 (29.7–34.3)	31.4 (29.4–33.6)	32.8 (30.1–36.4)	0.137
WC (cm)	101.8 (98.1–109.2)	101.2 (97.1–107.1)	105.1 (99.0–117.0)	0.091
WhtR	0.59 (0.56–0.63)	0.58 (0.56–0.61)	0.61 (0.57–0.67)	**0.044**
Total fat (kg)	36.14 (30.20–40.74)	34.63 (29.92–38.97)	37.95 (34.76–46.4)	0.071
Total fat (%)	37.48 (33.93–39.8)	36.22 (33.17–38.26)	39.31 (35.5–41.55)	**0.021**
Lean mass (kg)	58.40 (54.41–62.31)	58.56 (54.41–62.31)	57.88 (54.38–62.34)	0.866
Lean mass (%)	59.42 (57.11–62.85)	60.33 (58.62–63.69)	57.16 (55.55–60.73)	**0.009**
Trunk fat (kg)	19.75 (16.51–25.53)	18.50 (15.56–21.72)	21.50 (18.51–26.69)	**0.022**
Trunk fat (%)	55.86 (53.26–57.36)	55.28 (52.29–56.83)	57.07 (54.58–58.46)	**0.040**
FMI (kg/m^2^)	11.72 (10.47–13.18)	11.10 (10.23–12.74)	12.89 (11.39–15.51)	**0.034**
FFMI (kg/m^2^)	19.10 (18.2–20.26)	19.31 (18.20–20.18)	18.96 (18.23–20.81)	0.955

*Note:* Data are presented as median (p25–p75). Low VAI (≤ 2.52); high VAI (> 2.52); WhtR: waist–height ratio. Values in bold indicate statistical significance.

Abbreviations: BMI, body mass index; FFMI, fat‐free mass index; FMI, fat mass index; WC, waist circumference.

^∗^Age‐specific cutoff point of 2.52 based on Amato et al. [23].

^ⴕ^Low VAI vs. High VAI, Wilcoxon rank‐sum (Mann–Whitney) test.

**TABLE 2:** Blood pressure, metabolic and endocrine variables, and calculated indices of study participants, total sample, and by VAI cutoff^∗^.

	Total (*n* = 52)	Low VAI (*n* = 32)	High VAI (*n* = 20)	*p* value^ⴕ^
Arterial blood pressure				
SBP (mmHg)	120.8 (116.3–125.5)	120.0 (116.5–124.3)	121.5 (114.8–128.5)	0.873
DBP (mmHg)	68.3 (64.0–73.3)	66.5 (62.8–72.0)	70.0 (66.0–74.8)	0.096
Metabolic and hormones				
Glycemia (mg/dL)	96.0 (90.5–100.5)	97.0 (90.5–101.5)	94.0 (90.5–98)	0.200
Insulin (IU/mL)	18.0 (12.9–23.9)	14.9 (11.9–20.3)	22.9 (15.9–27.8)	**0.010**
Total‐C (mg/dL)	166.5 (144.0–187.5)	166.5 (146.0–188.5)	165.0 (140.5–180.5)	0.828
HDL‐C (mg/dL)	35.0 (31.0–40.0)	39.0 (35.0–46.0)	30.0 (26.0–33.5)	**0.000**
LDL‐C (mg/dL)	101.4 (84.1–123.1)	101.4 (84.8–127.6)	102.5 (81.9–118.0)	0.836
TG (mg/dL)	123.0 (94.0–153.5)	103.5 (82.5–121.5)	161.0 (146.5–179.0)	**0.000**
Uric acid (mg/dL)	2.88 (2.41–3.45)	2.8 (2.4–3.3)	3.05 (2.7–3.54)	0.121
CRP (mg/dL)	0.21 (0.11–0.41)	0.17 (0.07–0.45)	0.26 (0.15–0.38)	0.244
PAI‐1 (ng/mL)	42.06 (27.70–57.10)	31.94 (26.26–44.15)	51.61 (37.75–73.75)	**0.018**
Leptin (ng/mL)	27.74 (19.84–40.43)	25.41 (15.53–36.34)	29.3 (23.75–48.01)	0.083
Adiponectin (mg/mL)	2.73 (1.89–3.60)	3.20 (2.01–5.60)	2.09 (1.52–2.79)	**0.006**
Calculated indices				
HOMA‐IR	4.23 (3.03–5.81)	3.62 (2.90–5.10)	5.27 (3.95–6.52)	**0.020**
TyG	8.73 (8.43–8.90)	8.52 (8.29–8.73)	8.96 (8.85–9.04)	**0.000**
MSSS	0.59 (0.23–0.83)	0.26 (−0.01–0.51)	0.87 (0.80–1.12)	**0.000**
Ad/Lep	0.10 (0.06–0.17)	0.13 (0.07–0.21)	0.07 (0.03–0.11)	**0.002**
VAI	1.94 (1.37–2.80)	1.59 (1.20–1.84)	3.03 (2.68–3.67)	0.000

*Note:* Data are presented as median (p25–p75). total‐C: total cholesterol, HOMA‐IR: Homeostatic Model Assessment Insulin Resistance, TyG: triglyceride glucose index; Ad/Lep: adiponectin/leptin ratio. Values in bold indicate statistical significance.

Abbreviations: CRP, C‐reactive protein; DBP, diastolic blood pressure; LDL‐C, low‐density lipoprotein cholesterol; MSSS, metabolic syndrome severity score; PAI‐1, plasminogen activator inhibitor‐1; SBP, systolic blood pressure; VAI, visceral adiposity index.

^∗^Age‐specific cutoff point of 2.5 based on Amato et al. [23].

^ⴕ^Low VAI vs. High VAI, Wilcoxon rank‐sum (Mann–Whitney) test.

While body weight did not differ between VAI groups, WhtR, total fat, and trunk fat percentages were higher and lean mass percentage was lower in the H‐VAI group (Table 1). When expressed in kg, the H‐VAI group showed a trend toward a 10% higher total fat mass, and a significant 16% higher trunk fat mass, while lean mass did not differ. Accordingly, FMI was higher in H‐VAI, and FFMI did not differ between groups.

Total and LDL cholesterol did not differ between groups, and median values were within the normal range for the former and slightly elevated for the latter. Fasting blood glucose did not differ; however, the group with VAI‐defined adipose dysfunction (H‐VAI) had greater values of circulating insulin (Table 2), suggesting a state of insulin resistance that was confirmed by significantly higher HOMA‐IR and TyG values (Table 2). Circulating levels of PAI‐1 were 62% higher in the H‐VAI group, whereas adiponectin was 35% lower. The calculated MSSS was more than threefold higher in the H‐VAI group, and the adiponectin/leptin ratio (Ad/Lep), which was low and indicative of metabolic risk for both groups [32], was nearly 50% lower (Table 2).

**FIGURE 1:**
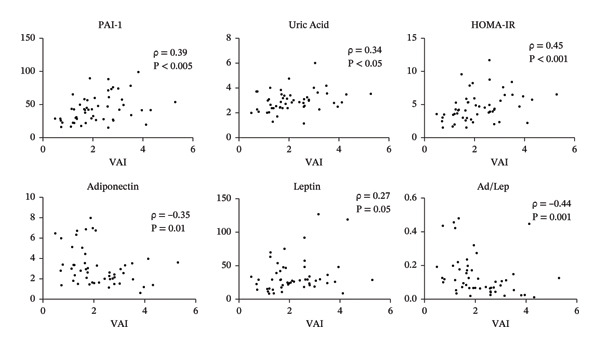
Associations between VAI and metabolism‐related variables: blood plasminogen activator inhibitor‐1 (PAI‐1, ng/mL) and uric acid (mg/dL), HOMA‐IR, adiponectin (μg/mL), leptin (ng/mL), and adiponectin/leptin ratio (Ad/Lep). Spearman’s correlation coefficients (rho) and values are shown for each correlation, *n* = 52.

Significant correlations were observed between VAI and relevant metabolic or endocrine parameters that are not included or directly related to parameters in VAI calculation. The index was directly correlated with PAI‐1, uric acid, and HOMA‐IR (with a strong trend for leptin) and inversely with adiponectin and Ad/Lep (Figure 1). To account for potential confounders, such as percent body fat (total and truncal) and physical activity (total METs min/week) [24], partial Spearman’s correlation via residual analysis was performed (Supporting Table S1). The associations between VAI and PAI‐1, HOMA‐IR, and adiponectin remained significant after adjusting for each potential confounder, suggesting that the associations were independent of these variables. Ad/Lep remained significant after adjusting for % truncal fat and METs, but not after adjusting for % total body fat, which is expected, given the close association between circulating leptin and adipose tissue.

Regarding the parameters that showed significant correlations with VAI (Figure 1), they were in turn also correlated with BMI and WC, except for PAI‐1 and adiponectin (which showed trends) (Supporting Table S2). WhtR correlated with all variables; however, the associations with PAI‐1, HOMA‐IR, and adiponectin were not as strong as those observed with VAI (Supporting Table S2 and Figure 1).

## 4. Discussion

Our results indicate that in nondiabetic young males with overweight and obesity, VAI is a valuable tool to assess metabolic health and adipose tissue functionality. The proposed cutoff point of 2.52 for this age group [23] allowed for the definition of two groups with significant differences in parameters of cardiometabolic health, indicating that it is a useful proxy to assess metabolic risk levels in young adults with excess weight.

It is widely described that it is not the amount of total fat mass present in an individual, but rather its functionality that determines cardiovascular and metabolic risk associated with excess weight [18–20]. Interestingly, while body weight or BMI did not differ between groups, body composition did, with higher total and central percent fat mass and lower percent lean mass in the H‐VAI group. Notably, FMI based on body composition assessment by DXA was elevated in H‐VAI; however, FFMI did not differ, supporting the ability of VAI to sense the contribution of body composition (specifically, fat) in cardiometabolic risk, which provides this index with an important advantage over BMI.

Insulin resistance, a hallmark of metabolic derangement and a relevant risk factor for type 2 diabetes, was significantly elevated in the H‐VAI group. This was valid for HOMA‐IR and TyG, both of which are highly correlated with the hyperinsulinemic–euglycemic clamp [33, 34], the “gold standard” method, that is, however, an invasive and expensive procedure. Based on the HOMA‐IR threshold of 4.65 to define insulin resistance in subjects with BMI > 28.9 kg/m^2^ [35], our VAI‐defined groups are consistent with discriminating the healthier from the unhealthier phenotype. However, TyG has emerged as a highly sensitive surrogate for insulin resistance assessment and metabolic syndrome predictor [34, 36], given that TG accumulate in muscles and lead to intramyocellular insulin resistance [27]. This index is based on even more accessible parameters than HOMA‐IR, and numerous reports indicate that it is better in its capacity to predict metabolic syndrome [36] and can also reflect type 2 diabetes and cardiovascular diseases [37, 38]. TyG values in our study agree with previous reports assessing its predictive capacity for the presence or absence of metabolic syndrome, with the median of the H‐VAI group above, and the L‐VAI group below the proposed TyG cutoff of 8.80 for adult populations [36].

It is extensively documented that adipose tissue dysfunction has a relevant role in the development of obesity‐related diseases, such as metabolic syndrome [20]. Nevertheless, given that metabolic syndrome diagnosis is based on a dichotomic classification (presence or absence) [39], limitations arise as the criterion is not sensitive to improvements in particular features, unless they achieve the corresponding cutoff value. The MSSS clusters risk factors into a continuous variable [28], which is useful to address more subtle differences or changes. The score can be interpreted as a *Z*‐score, with higher values indicating elevated risk, particularly above 1. The H‐VAI group showed a significantly higher MSSS and was also close to this threshold value, indicating a high consistency between assessments.

Our results indicate that VAI‐defined adipose tissue dysfunction is not only able to reflect metabolic derangements, but also adipose endocrine dysfunction as well. The Ad/Lep is an emerging indicator of endocrine imbalance, dysfunctional adipose tissue, and inflammation [32, 40], which shows a robust correlation with insulin resistance and other alterations, more so than each adipokine alone [41]. According to previously proposed cutoff values of Ad/Lep [32] based on a population that included BMIs ranging from normal weight to obesity, values below 0.5 suggest severe risk, which is the case in both groups in this study. However, our sample includes only participants with excess weight, which may account for these overall high values, given that leptin is in general elevated [42] and adiponectin is decreased [43] as BMI increases. To the best of our knowledge, there are no previous studies reporting Ad/Lep values that may discriminate between metabolically healthier vs unhealthier subjects within participants with overweight and obesity. Our sample of young males showed a near 50% decrease in this index in H‐VAI as compared with L‐VAI, again supporting the relevance of VAI, in this case reflecting differences in adipose tissue endocrine functionality within a range of BMI restricted to those with excess weight.

In the search for markers of dysfunctional adipose tissue, metabolome analysis has been proposed as a metabolic health predictor among subjects with obesity [44]. Cirulli et al. showed that metabolite levels are not only able to predict obesity, but in BMI‐matched subjects, a specific metabolome signature is determined by differences in metabolic health [44]. In their study, uric acid was the most salient factor differentially detected in the metabolome of subjects with obesity, also showing higher levels in subjects with the same BMI but worse overall metabolome profile and cardiometabolic health. Besides the widely known link between hyperuricemia and gout, elevated circulating uric acid is also associated with hepatic steatosis [45] and the components of metabolic syndrome [46]. Moreover, uric acid levels are strongly associated with visceral and hepatic but not subcutaneous fat [47] and have been negatively associated with adiponectinemia [48]. In our young male population, mean values for uric acid were below the cutoff relating hyperuricemia with metabolic syndrome in adults (5.6 mg/dL) [49]. Even though median uric acid values in this study did not differ between H‐VAI vs L‐VAI groups, our observations show a significant positive correlation between uric acid and VAI, possibly indicating an early alert worth paying attention to, given its proposed role as a modifiable risk factor [50].

Circulating levels of the acute‐phase protein PAI‐1, which has been related to diabetes, inflammation, metabolic syndrome, and cardiovascular risk [51, 52], were also elevated in our sample of young males with H‐VAI. PAI‐1 is expressed and secreted by adipose tissue, mainly the visceral depot [53, 54]. In adipose dysfunctionality, the proinflammatory environment is implicated in the regulation and secretion of PAI‐1. Exposure of adipose tissue explants, *in vitro* differentiated adipose cells, and primary adipocytes to exogenous tumor necrosis factor alpha (TNF‐α) or transforming growth factor beta (TGF‐β) increases protein and mRNA expression of PAI‐1 [54, 55], thus exacerbating adipose dysfunctionality under inflammatory conditions. Serum PAI‐1 levels have been directly correlated with those of uric acid in healthy subjects [48]. The ability of the VAI‐based classification to reveal differences in circulating PAI‐1 is another evidence of the importance of this index in discriminating overall metabolic health parameters among subjects with excess weight.

Limitations of our study include the relatively low number of participants, which precluded us from performing more complex statistical modeling to robustly address potential confounders. In addition, only young males were included. Our purpose was to assess the potential of VAI classification in this homogeneous group of otherwise healthy participants with excess weight, as a good starting point to address the ability of this index as an easy‐access tool to identify subjects at greater risk for developing cardiometabolic disorders. Given the sex and age limitations, the results should be interpreted with caution when considering different populations. Our results encourage future studies including females and wider age ranges, including children, adolescents, and aging populations. In addition, our study design included only the assessment of fasting blood parameters, and future studies are encouraged to incorporate postprandial evaluations to have a broader appraisal of the participants’ metabolic health. Another interesting future direction would be to determine the value of VAI in predicting risk in populations with BMI within the normal range. The “normal weight obese” phenotype is a relevant group that despite not meeting the BMI‐based obesity criterion shows elevated cardiometabolic risk; however, they may go unnoticed and untreated, increasing the risk for morbidity and mortality [56].

## 5. Conclusions

Our findings support the relevance and usefulness of VAI as a proxy for several aspects of adipose tissue functionality and cardiometabolic risk in young adult males. Its strong correlations with relevant metabolites, hormones, and health indices provide information on metabolic health, independent of the classification of overweight or obesity by BMI. The use of an index based on rather accessible assessments, such as VAI, has an important value for the clinical practice and public health. For any given BMI that defines overweight or obesity, knowledge of adipose tissue functionality may aid in triaging patients to make ethical and personalized treatment decisions. This information may determine the need for immediate action for weight loss or may reflect a healthy lifestyle and a metabolically healthy profile, in which pressing weight loss advice may induce unhealthy weight control practices, psychological distress, deleterious effects on insulin sensitivity [57], or a lower overall impact [58]. In the latter case, the most adequate advice may be periodic medical checkups and encouragement to continue with a healthy lifestyle. Given the simplicity of its calculation using rather easily accessible parameters, we encourage the use of VAI and further research into its value as a tool to better assess metabolic health in different populations with normal weight, overweight, and obesity.

## Author Contributions

Study concept and design: Mariana Cifuentes; acquisition of data: Gabriela Yuri, Natalia Santillana, and Liliana Soto; analysis and interpretation of data: Mariana Cifuentes, Camila Corvalán, and Ana Pereira; drafting of the manuscript: Mariana Cifuentes, Gabriela Yuri, Natalia Santillana, and Liliana Soto; critical revision of the manuscript: Ana Pereira and Camila Corvalán; statistical analysis: Mariana Cifuentes; obtained funding: Mariana Cifuentes, Gabriela Yuri, Natalia Santillana, and Ana Pereira; administrative, technical, or material support: Ana Pereira and Camila Corvalán; and study supervision: Mariana Cifuentes, Ana Pereira, and Camila Corvalán.

## Funding

This work was supported by the following grants from Agencia Nacional de Investigación y Deasarrollo (ANID): Fondecyt program (grant number 1211477), Fondap Program (grant number 15130011) and Fondap Apoyo Program (grant number 1523A0008) to Mariana Cifuentes, Fondecyt program (grant number 1230813) to Ana Pereira, Beca Doctorado Nacional (grant number 21220077) to Gabriela Yuri, and Beca Doctorado Nacional (grant number 212304016) to Natalia Santillana.

## Conflicts of Interest

The authors declare no conflicts of interest.

## Supporting Information

The following supporting information is available with this article:

Table S1: Adjusted and unadjusted Spearman corelations of variables that showed significant correlations with VAI (*n* = 52).

Table S2. Spearman corelations of body mass index (BMI), waist circumference (WC) and waist‐height ratio (WhtR) with variables that showed significant correlations with VAI (*n* = 52).

## Supporting information


**Supporting Information** Additional supporting information can be found online in the Supporting Information section.

## Data Availability

The data that support the findings of this study are available from the corresponding author upon reasonable request.
