# Supplementary material for: Association Between Metabolic and Endocrine Parameters With Visceral Adiposity Index–Defined Adipose Tissue Dysfunction in Young Adult Males
Source: J Obes. 2026 Mar 28;2026:5233463. doi: 10.1155/jobe/5233463 (PMC13140360; doi:10.1155/jobe/5233463)
Supplement: Supplementary file 1 — Supporting Information Additional supporting information can be found online in the Supporting Information section. [file JOBE-2026-5233463-s001.docx]

**Yuri et al. Supplementary Tables.**

**Suppl. Table S1. Adjusted and unadjusted Spearman corelations of variables that showed significant correlations with VAI (n=52)**

|  | **Adjustment variable** | | | | | | | |
| --- | --- | --- | --- | --- | --- | --- | --- | --- |
|  | **None** | | **% total fat** | | **% trunk fat** | | **Total METs**  **(min-wk)** | |
|  | Rho | P-value | Rho | P-value | Rho | P-value | Rho | P-value |
| PAI-1 | 0.39 | **<0.005** | 0.29 | **<0.05** | -0.36 | **<0.01** | 0.41 | **<0.005** |
| Uric Acid | 0.34 | **<0.05** | 0.24 | 0.09 | 0.18 | 0.20 | 0.33 | **<0.05** |
| HOMA-IR | 0.45 | **<0.001** | 0.28 | **<0.05** | 0.42 | **0.002** | 0.44 | **0.001** |
| Adiponectin | -0.35 | **0.01** | -0.34 | **0.01** | -0.31 | **<0.05** | -0.35 | **0.01** |
| Leptin | 0.27 | 0.05 | -0.04 | 0.77 | 0.21 | 0.13 | 0.25 | 0.07 |
| Ad/Lep | -0.44 | **0.001** | -0.24 | 0.08 | -0.36 | **<0.01** | -0.44 | **0.001** |

VAI: visceral adipose index; PAI-1: plasminogen activator inhibitor-1, HOMA-IR: homeostatic model assessment insulin resistance; Ad/Lep: adiponectin/ leptin ratio.

**Suppl. Table S2. Spearman corelations of body mass index (BMI), waist circumference (WC) and waist-height ratio (WhtR)with variables that showed significant correlations with VAI (n=52)**

|  | **BMI** | | **WC** | | **WhtR** | |
| --- | --- | --- | --- | --- | --- | --- |
|  | Rho | P-value | Rho | P-value | Rho | P-value |
| PAI-1 | 0.18 | 0.211 | 0.26 | 0.059 | 0.30 | **0.034** |
| Uric Acid | 0.41 | **0.003** | 0.38 | **0.005** | **0.41** | **0.003** |
| HOMA-IR | 0.51 | **<0.001** | 0.46 | **<0.001** | **0.40** | **0.003** |
| Adiponectin | -0.24 | 0.089 | -0.23 | 0.095 | -0.28 | **0.042** |
| Leptin | 0.52 | **<0.001** | 0.56 | **<0.001** | **0.55** | **<0.001** |
| Ad/Lep | -0.51 | **<0.001** | -0.54 | **<0.001** | **-0.56** | **<0.001** |

VAI: visceral adipose index; PAI-1: plasminogen activator inhibitor-1, HOMA-IR: homeostatic model assessment insulin resistance; Ad/Lep: adiponectin/ leptin ratio.
